# Downregulation of CTRP-3 by Weight Loss In Vivo and by Bile Acids and Incretins in Adipocytes In Vitro

**DOI:** 10.3390/ijms21218168

**Published:** 2020-10-31

**Authors:** Andreas Schmid, Jonas Gehl, Miriam Thomalla, Alexandra Hochberg, Anja Kreiß, Marissa Patz, Thomas Karrasch, Andreas Schäffler

**Affiliations:** Department of Internal Medicine III, Giessen University Hospital, 35392 Giessen, Germany; jgehl47@gmail.com (J.G.); miriam.thomalla@innere.med.uni-giessen.de (M.T.); alexandra.hochberg@innere.med.uni-giessen.de (A.H.); anja.f.kreiss@med.uni-giessen.de (A.K.); marissa.patz@med.uni-giessen.de (M.P.); thomas.karrasch@innere.med.uni-giessen.de (T.K.); andreas.schaeffler@innere.med.uni-giessen.de (A.S.)

**Keywords:** C1q/TNF-related protein-3, obesity, bariatric surgery, low calorie diet, bile acids, incretins

## Abstract

The adipokine CTRP-3 (C1q/TNF-related protein-3) exerts anti-inflammatory and anti-diabetic effects. Its regulation in obesity and during weight loss is unknown. Serum and adipose tissue (AT) samples were obtained from patients (*n* = 179) undergoing bariatric surgery (BS). Moreover, patients (*n* = 131) participating in a low-calorie diet (LCD) program were studied. CTRP 3 levels were quantified by ELISA and mRNA expression was analyzed in AT and in 3T3-L1 adipocytes treated with bile acids and incretins. There was a persistent downregulation of CTRP-3 serum levels during weight loss. CTRP-3 expression was higher in subcutaneous than in visceral AT and serum levels of CTRP-3 were positively related to AT expression levels. A rapid decrease of circulating CTRP-3 was observed immediately upon BS, suggesting weight loss-independent regulatory mechanisms. Adipocytes CTRP-3 expression was inhibited by primary bile acid species and GLP 1. Adipocyte-specific CTRP-3 deficiency increased bile acid receptor expression. Circulating CTRP-3 levels are downregulated during weight loss, with a considerable decline occurring immediately upon BS. Mechanisms dependent and independent of weight loss cause the post-surgical decline of CTRP-3. The data strongly argue for regulatory interrelations of CTRP-3 with bile acids and incretin system.

## 1. Introduction

Among the increasing number of secretory proteins that have been identified as so-called adipo(cyto)kines, the family of C1q/TNF-related proteins (CTRPs) comprises 15 distinct adiponectin paralogs [1]. CTRPs are expressed and secreted by various tissues and cell-types as endocrine factors, affecting a broad panel of physiological processes at different anatomical localizations [1]. CTRP-3 as a prominent and pleiotropic CTRP is mainly expressed in adipose tissue but also in gonads and kidney [2]. It represents a mainly anti-inflammatory adipokine in the context of cell proliferation [3,4,5], metabolism [6,7], and inflammation [8,9,10]. Importantly, CTRP-3 regulates hepatic carbohydrate and lipid metabolism [6,11] as well as adipogenesis [8]. Further, it has immunomodulatory properties with anti-inflammatory effects locally in adipose tissue [8,9,12] but also systemically [7,9]. Therefore, CTRP-3 represents a putative regulatory element linking metabolism and inflammation (“metaflammation”). Similar to adiponectin, a negative correlation of systemic CTRP-3 levels with the occurrence of obesity and type 2 diabetes mellitus (T2D) has repeatedly been reported [13,14,15]. However, circulating levels were not affected in short term by an oral lipid load or during an oral glucose tolerance test in subjects from a cross-sectional study cohort [16]. Of note, a recent study presented gender-specific effects, indicating that the reciprocal correlation of serum CTRP-3 with obesity might be specific for females [17]. Because of the observed negative associations with overweight and obesity and the beneficial metabolic properties, CTRP-3 might be considered both as a potential biomarker and future drug target in the context of the metabolic syndrome, especially in obesity and T2D. Therefore, data on local CTRP-3 gene expression in adipose tissue compartments in obesity and on the regulation of systemic CTRP-3 during weight loss is of physiological importance.

Obesity as a common and challenging public health issue is being addressed by different therapeutic approaches. In addition to conservative management including medication, dietary and general life style interventions, bariatric surgery techniques are increasingly applied. Currently, Roux-en-Y gastric bypass (RYGB) is a most frequently applied option, combining the principles of food-intake restriction and malabsorption [18,19]. The alteration of gastrointestinal anatomy regularly induces hormonal changes (incretins) and a modification of bile acid physiology. A significant and rapid increase of systemic bile acids following RYGB [20,21,22] is observed regularly. Interestingly, lower circulating bile acid concentrations have been reported in obese patients when compared to lean subjects [20,21]. Post-surgical modification of serum bile acid quantities or bile acid subspecies might represent a novel and weight loss-independent mechanism mediating metabolic improvements. In this context, it needs to be clarified whether CTRP-3 is altered upon bariatric surgery and whether it is regulated by bile acids in adipocytes in vitro.

A large and well-characterized obesity study cohort (Research in Obesity and Bariatric Surgery, ROBS) has been established at the University Obesity Center, Giessen, Germany [23]. The ROBS study comprises obese patients undergoing either bariatric surgery (*n* = 179; RYGB or gastric sleeve (GS)) or life-style intervention including a low-calorie formula diet (LCD; *n* = 131).

Our underlying and unifying hypothesis indicates that CTRP-3 is regulated by weight loss and that its expression in adipocytes might be influenced by bile acids and incretins. Bile acids and incretins are the two main physiological systems that change rapidly and persistently upon bariatric surgery.

The aim of the present study was to investigate:

- the detailed CTRP-3 mRNA expression profile in subcutaneous and visceral adipose tissue samples obtained from severely obese patients undergoing bariatric surgery,
- basal CTRP-3 serum concentrations in obese patients and their changes after bariatric surgery or during LCD,
- correlations of adipose tissue CTRP-3 gene expression with its circulating protein concentrations and with a broad panel of anthropometric and biochemical parameters by univariate and multivariate analysis,
- effects of bile acids and incretins on CTRP-3 expression in adipocytes in vitro.

## 2. Results

### 2.1. Study Populations

A total of 179 patients were included in the bariatric surgery study. Serum samples and visceral/subcutaneous adipose tissue specimens were available in all of them. Overall, the patients (males: 37; females: 142) were severely obese with a mean body mass index (BMI) of 53.3 ± 6.9 kg/m^2^ and a range between 40.2 to 83.7 kg/m^2^. Mean age was 39.8 ± 11.2 years.

The patients of the cohort undergoing a low-calorie formula diet (*n* = 131; males: 43; females: 88) had a mean BMI of 43.5 ± 6.7 kg/m^2^ with a range from 29.7 to 81.5 kg/m^2^. The mean age in this cohort was 42.1 ± 12.0 years.

### 2.2. Baseline CTRP-3 Serum Concentrations and CTRP-3 Gene Expression in Adipose Tissue of Obese Subjects

**Figure 1:**
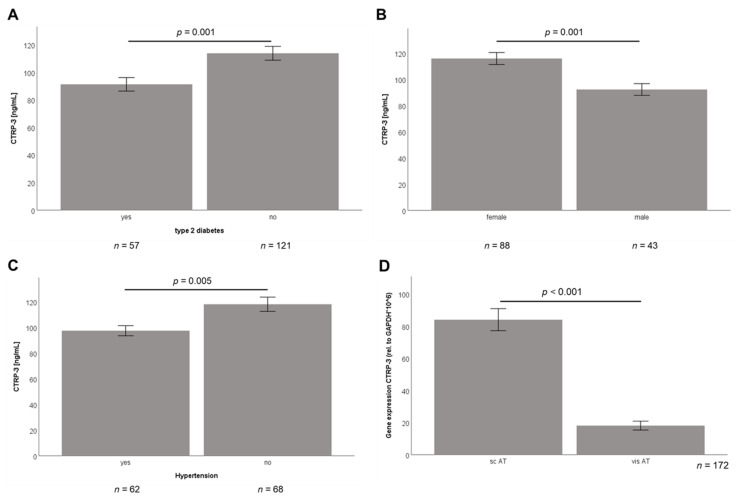
Baseline serum C1q/TNF-related protein-3 (CTRP-3) concentrations in patient cohorts of bariatric surgery and low-calorie formula diet. CTRP-3 serum concentrations were measured by ELISA. AT, adipose tissue; BS, bariatric surgery; GAPDH, glyceraldehyde-3-phosphate dehydrogenase; LCD, low-calorie formula diet; sc, subcutaneous; vis, visceral. (**A**) CTRP-3 serum levels are decreased in BS patients with diagnosed type 2 diabetes. (**B**) CTRP-3 serum levels are higher in females when compared to male patients within the LCD cohort. (**C**) CTRP-3 serum levels are lower in LCD patients with hypertension. (**D**) CTRP-3 expression is significantly higher in subcutaneous adipose tissue when compared to visceral adipose tissue in BS patients.

In the subgroup of bariatric surgery patients (*n* = 179), serum CTRP-3 levels ranged from 13.5 to 389.7 ng/mL with a mean concentration of 106.5 ± 51.0 ng/mL. CTRP-3 concentrations were positively correlated with BMI (r = 0.16; *p* = 0.038) and percentage body fat (r = 0.18; *p* = 0.024). Patients with diagnosed T2D (*n* = 57) had lower CTRP-3 levels than non-diabetic individuals (*n* = 121) as displayed in Figure 1A (*p* = 0.001). There was a slight difference in CTRP-3 serum concentrations between genders with a trend to higher levels in female subjects, yet without statistical significance (*p* = 0.102).

Patients participating in the low-calorie formula diet program had serum CTRP-3 concentrations ranging from 39.6 to 317.1 ng/mL with a mean of 108.1 ± 40.5 ng/mL. Female subjects within this cohort exhibited considerably higher CTRP-3 levels than males (*p* = 0.001) (Figure 1B). The trend to lower levels in T2D patients reported above did not reach statistical significance (*p* = 0.114) in this cohort (data not shown). Furthermore, decreased CTRP-3 serum concentrations were observed in patients with hypertension (*p* = 0.005) (Figure 1C).

Table A1 (see Appendix) provides a comparative summary of mean serum CTRP-3 levels in the present study and in studies of obese patients published earlier [13,14,24]. This might be useful for the reader since different ELISA kits are currently being used. For a critical interpretation of data, the respective ELISA kits are mentioned in the figure legend.

In the bariatric surgery group, paired adipose tissue specimens were resected from subcutaneous and visceral compartments and gene expression analysis was applied in 172 patients. As shown in Figure 1D, CTRP-3 mRNA levels were significantly higher in subcutaneous adipose tissue when compared to visceral adipose tissue (*p* < 0.001). To the best of our knowledge, this is the largest cohort of patients that has been investigated for adipose tissue CTRP-3 gene expression.

### 2.3. Correlation Analysis of Baseline Data

The Spearman test was applied in order to identify significant correlations of basal serum CTRP-3 concentrations with anthropometric and biochemical parameters in bariatric and LCD patients (Table 1A,B).

**Figure 2:**
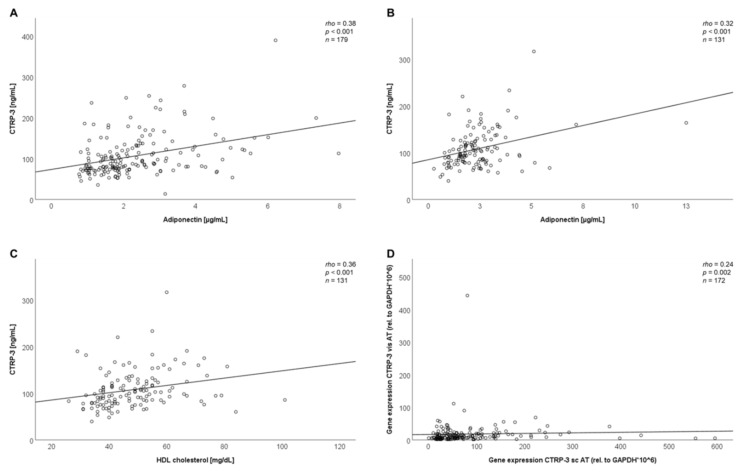
Correlation of baseline CTRP-3 serum levels with anthropometric and biochemical parameters. Adiponectin and CTRP-3 serum concentrations were measured by ELISA. Gene expression levels in adipose tissues were analyzed via RT-PCR. AT, adipose tissue; BS, bariatric surgery; GAPDH, glyceraldehyde-3-phosphate dehydrogenase; LCD, low calorie formula diet; sc, subcutaneous; vis, visceral. (**A**) Positive correlation of CTRP-3 and adiponectin serum concentrations within the BS cohort. (**B**) Positive correlation of CTRP-3 and adiponectin serum concentrations within the LCD cohort. (**C**) Positive correlation of serum CTRP-3 and HDL levels within the LCD cohort. (**D**) Positive correlation of CTRP-3 gene expression in subcutaneous and visceral adipose tissue of bariatric patients.

In the bariatric surgery group, positive correlations of CTRP-3 serum concentrations with adiponectin (rho = 0.38; *p* < 0.001) (Figure 2A) and leptin levels (rho = 0.16; *p* = 0.033) (Table 1A) were found. The strong correlation with adiponectin remained significant even after correction for BMI (data not shown). Furthermore, circulating CTRP-3 concentrations were significantly correlated with subcutaneous (rho = 0.23; *p* = 0.003) and visceral adipose tissue CTRP-3 gene expression (rho = 0.16; *p* = 0.033) (Table 1A). CTRP-3 mRNA levels in subcutaneous and visceral adipose tissue were correlated positively with each other (*p* = 0.002; rho = +0.24), as it is depicted in Figure 2D.

Among LCD patients, CTRP-3 serum levels also were positively correlated with adiponectin (rho = 0.32; *p* < 0.001) (Figure 2B) and leptin concentrations (rho = 0.23; *p* = 0.007; Table 1B) with both correlations remaining significant even after correction for BMI. Of note, a positive correlation between serum CTRP-3 and serum HDL was observed (rho = 0.36; *p* < 0.001) (Figure 2C).

### 2.4. Significant Decline of CTRP-3 Serum Concentrations After Intervention

**Figure 3:**
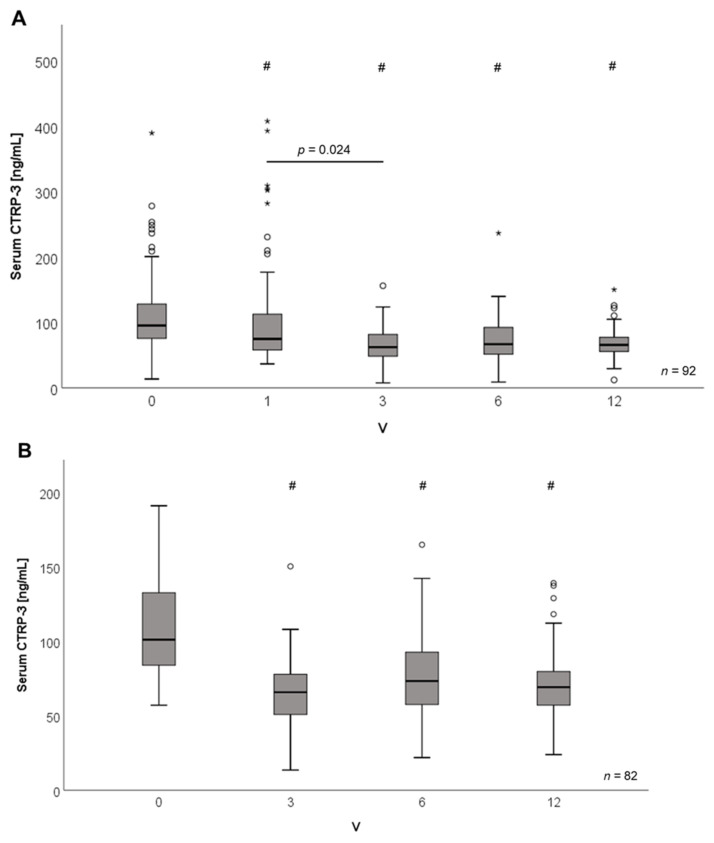
CTRP-3 serum concentrations decrease during weight loss induced by bariatric surgery or low-calorie diet, respectively. CTRP-3 serum concentrations were measured by ELISA. BS, bariatric surgery; LCD, low calorie formula diet; study points: V0, start of intervention; V1, 3 days post-surgery; V3, V6, V12 = 3, 6, 12 months after start of intervention. (**A**) Rapid and sustained decline of circulating CTRP-3 levels upon bariatric surgery. Outliers; * extreme values; ^#^ *p* < 0.001 when compared to V0; *p* = 0.024 between V1 and V3. (**B**) Sustained decline of circulating CTRP-3 levels during LCD. *, outliers; ^#^ *p* < 0.001 when compared to V0.

Bariatric surgery and LCD resulted in a highly significant weight loss at V12 when compared to baseline levels, as it was reported earlier for a smaller subgroup of the same study cohort [23]. On average, bariatric surgery patients lost 56.5 ± 17.1 kg and LCD patients lost 30.3 ± 15.6 kg. Of note, a significant (*p* < 0.001) decline of serum CTRP-3 concentrations by ~10% occurred rapidly within three days upon bariatric surgery (study point V1; Figure 3A). Within 3 months, CTRP-3 levels declined by ~36% from 106.46 down to 68.12 ng/mL (*p* < 0.001). During the sustained decrease, serum CTRP-3 quantities remained rather constant at ~65% of baseline levels during the following 12 months (Figure 3A). This reduction of CTRP-3 levels was observed independently of the surgical intervention technique applied (i.e., RYGB versus sleeve gastrectomy).

Similarly, a rapid and considerable decline (*p* < 0.001) of circulating CTRP-3 by ~38% from baseline levels of 108.10 down to 67.18 ng/mL occurred after 3 months in patients participating in LCD. On average, this decline sustained throughout the visits V3, V6, and V12 (Figure 3B).

### 2.5. Correlation Analysis of Adipose Tissue CTRP-3 and Bile Acid Receptor Gene Expression

If early changes in bile acid metabolism upon bariatric surgery would be responsible for the weight loss-independent and rapid decline of CTRP-3, the gene expression of bile acid receptors in adipose tissue is of interest. Correlation analysis was applied for gene expression levels of CTRP-3 and the bile acid receptor TGR5 in visceral and subcutaneous adipose tissue (TGR5 gene expression data were published in detail [25]). No significant correlation between CTRP-3 expression and TGR5 expression in visceral (r = 0.04; *p* = 0.78) and in subcutaneous adipose tissue (r = −0.10; *p* = 0.53) was found. FXR mRNA quantities in adipose tissue had earlier been found to be too low in humans for a valid quantification via RT-PCR [25].

### 2.6. Bile Acid Sub-Species Exert Differential Effects on CTRP-3 mRNA Expression in Adipocytes In Vitro

**Figure 4:**
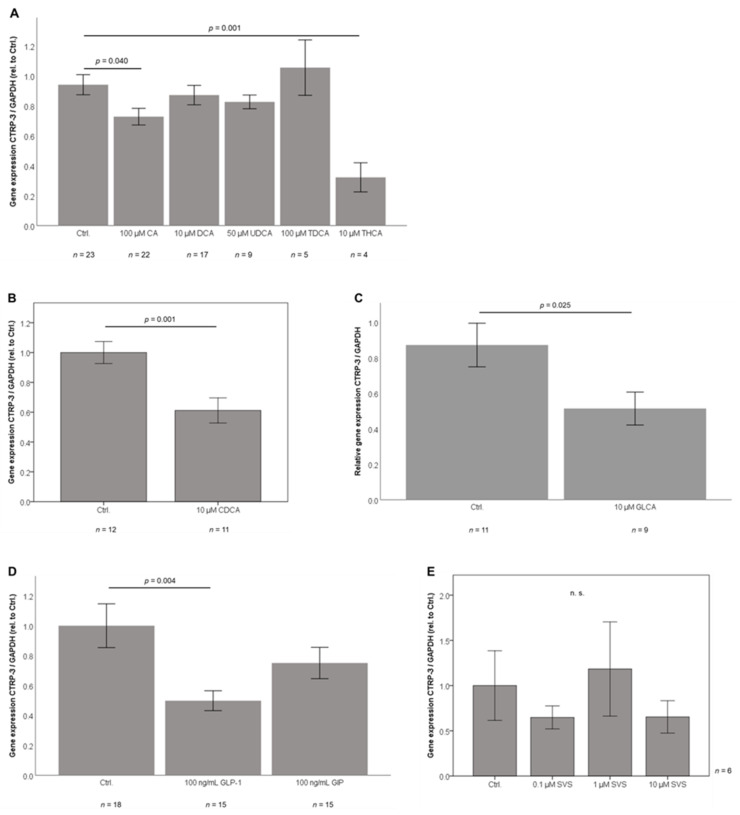
CTRP-3 gene expression is affected by bile acids and incretins in adipocytes in vitro. 3T3-L1 adipocytes were stimulated with bile acids, incretins and the cholesterol inhibitor simvastatin. CTRP-3 gene expression was analyzed by real-time PCR. BS, bariatric surgery; CA, cholic acid; CDCA, chenodeoxycholic acid; Ctrl., control; DCA, deoxycholic acid; GAPDH, glyceraldehyde-3-phosphate dehydrogenase; GIP, glucose-dependent insulinotropic polypeptide; GLCA, glycolithocholic acid; GLP-1, glucagon-like peptide 1; LCD, low calorie formula diet; SVS, simvastatin; TDCA, taurodeoxycholic acid; THCA, taurohyocholic acid, TLCA, taurolithocholic acid; UDCA, ursodeoxycholic acid. (**A**) Downregulation of adipocyte CTRP-3 mRNA expression by CA and THCA. (**B**) CDCA inhibits CTRP-3 gene expression in adipocytes. (**C**) GLCA downregulates CTRP-3 gene expression in adipocytes. (**D**) GLP-1 downregulates CTRP-3 gene expression in adipocytes. (**E**) Adipocyte CTRP-3 mRNA levels are unaffected by simvastatin.

Since total bile acid concentrations rapidly increase upon bariatric surgery, bile acid-sub-species are of interest [25] and might represent molecular mediators of CTRP-3 expression in adipocytes. Thus, mature 3T3-L1 adipocytes were stimulated with a panel of different bile acid sub-species in non-cytotoxic doses that had been shown to be effective but not toxic on adipokine expression levels in prior experiments [25]. CTRP-3 gene expression was significantly downregulated by the primary bile acids cholic acid (*p* = 0.04; Figure 4A) and chenodeoxycholic acid (*p* = 0.001; Figure 4B). Taurohyocholic acid caused a strong suppression (*p* = 0.001; Figure 4A) of CTRP-3. CTRP-3 mRNA levels were unresponsive to treatment with the secondary bile acids deoxycholic acid (Figure 4A), ursodeoxycholic acid (Figure 4A), and taurodeoxycholic acid (Figure 4A). On the other hand, the secondary bile acid glycolithocholic acid significantly downregulated CTRP-3 gene expression (Figure 4C), whereas the taurine-conjugated molecule taurolithocholic acid had no effect (data not shown). Thus, we identified a significant and inhibitory effect of specific bile acid sub-species on adipocyte CTRP-3 gene expression for the first time. This effect might depend on the molecular structure and the respective amino acid conjugation of bile acids.

In separate experiments, the mRNA levels of classical bile acid receptors such as FXR and TGR5 were analyzed in 3T3-L1 adipocytes upon stimulation with exogenous, recombinant CTRP-3 (expressed in H5 insect cells; 10 µg/mL). Gene expression levels of both receptors were found to be unresponsive to CTRP-3 treatment (data not shown).

### 2.7. GLP-1 Treatment Downregulates CTRP-3 Gene Expression in Adipocytes

Besides bile acids, incretin hormones are modulated upon bariatric surgery and might represent regulating factors of adipose tissue gene expression. Thus, mature 3T3-L1 adipocytes were incubated with high doses of GLP-1 or GIP, respectively (each 100 nM). GLP-1 induced a significant decline of CTRP-3 mRNA expression by about 50% (*p* = 0.001; Figure 4D). On the other hand, cells treated with GIP showed a slightly decreased CTRP-3 expression (~−25%), but this trend was statistically not significant (Figure 4D).

### 2.8. Adipocyte CTRP-3 mRNA Expression is Unresponsive to Simvastatin

Given the observed correlation of circulating CTRP-3 with HDL cholesterol levels (suggesting a potential link to cholesterol metabolism), 3T3-L1 adipocytes were treated with a dose spectrum of simvastatin (0.1, 1, and 10 µM), a pharmacological inhibitor of cholesterol synthesis (inhibition of the enzyme 3-hydroxy-3-methylglutaryl-coenzym-A-reductase). Independent of the applied doses, no significant effects of simvastatin on CTRP-3 expression were observed (Figure 4E).

### 2.9. CTRP-3 and Bile Acid Receptor Gene Expression in the Murine System

**Figure 5:**
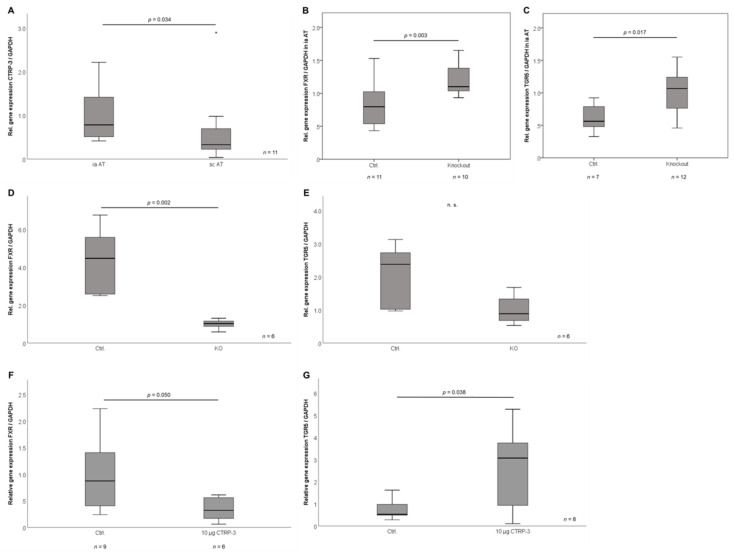
CTRP-3 and bile acid receptor expression in adipose tissue in murine models in vivo. CTRP-3 and bile acid receptor (FXR, TGR5) gene expression levels in murine adipose tissues and primary adipocytes (derived from adipose progenitor cells isolated from adipose tissue) were analyzed by RT-PCR. AT, adipose tissue; ia, intra-abdominal; Ctrl., control mice; FXR, farnesoid x receptor; GAPDH, glyceraldehyde-3-phosphate dehydrogenase; sc, subcutaneous; KO, adipocyte-specific CTRP-3 knock out mice; TGR5, G-protein coupled bile acid receptor. (**A**) CTRP-3 mRNA levels in intra-abdominal and in subcutaneous AT of wildtype mice (C57BL/6J; age 6 months; *n* = 11). (**B**) Elevated gene expression of FXR in intra-abdominal AT of CTRP-3 knockout mice (age 6 months; *n* = 10–11). (**C**) Elevated gene expression of TGR5 in intra-abdominal AT of CTRP-3 knockout mice (age 6 months; *n* = 7–12). (**D**) Decreased gene expression of FXR in primary adipocytes derived from subcutaneous AT of CTRP-3 knockout mice (*n* = 6). (**E**) TGR5 gene expression in primary adipocytes derived from subcutaneous AT of CTRP-3 knockout mice (*n* = 6). (**F**) Downregulation of FXR gene expression in intra-abdominal AT of wildtype mice (C57BL/6J; age 10 weeks; *n* = 5–9) by intraperitoneal injection of recombinant CTRP-3. (**G**) Induction of TGR5 gene expression in intra-abdominal AT of wildtype mice (C57BL/6J; age 10 weeks; *n* = 5–9) by intraperitoneal injection of recombinant CTRP-3.

CTRP-3 mRNA levels were analyzed in intra-abdominal and subcutaneous adipose tissue of male wildtype C57BL/6 mice. There were higher expression levels in intra-abdominal than in subcutaneous adipose tissue (*p* = 0.034; *n* = 11; Figure 5A). This stands in contrast to significantly higher subcutaneous than visceral CTRP-3 expression levels observed in human samples (Figure 1E).

In order to test the upcoming hypothesis of regulatory interactions between CTRP-3 and bile acids, gene expression levels of the bile acid receptors FXR and TGR5 were analyzed in intra-abdominal adipose tissue of adipocyte-specific CTRP-3 knockout mice. Most interestingly, both FXR and TGR5 expression were considerably up-regulated in knockout versus wildtype mice (*p* = 0.003 and *p* = 0.017; Figure 5B,C).

In a further approach, primary intra-abdominal and subcutaneous pre-adipocytes from female CTRP-3 knockout and control mice were cultured and differentiated to mature adipocytes ex vivo. While mRNA levels of bile acid receptors were not affected by CTRP-3 knockout in intra-abdominal adipocytes (data not shown), gene expression analysis revealed decreased FXR expression in subcutaneous adipocytes derived from CTRP-3 knockout mice (*p* = 0.002; Figure 5D). For TGR5, a non-significant trend to lower mRNA levels in CTRP-3 KO was observed (*p* = 0.065; Figure 5E).

In a separate experimental setting, male wildtype C57BL/6 mice were intraperitoneally injected with recombinant CTRP-3 (from H5 cells; injection doses: 1 and 10 µg/animal) for an incubation time of 2.5 h and intra-abdominal adipose tissue samples were obtained post-mortem and analyzed for bile acid receptor mRNA expression. Most interestingly, divergent effects were observed: while FXR mRNA levels were downregulated by application of 10 µg CTRP-3 (*p* = 0.050; Figure 5F), TGR5 expression was strongly induced (*p* = 0.038; Figure 5G).

## 3. Discussion

Besides its immuno-modulating functions as a potent LPS-antagonist in adipocytes and adipose tissue inflammation [8,9,12], the beneficial effects of CTRP-3 on glucose and lipid metabolism have been characterized in recent years [6,11]. Most interestingly and similar to adiponectin, systemic CTRP-3 levels have repeatedly been described to be lower in subjects suffering from obesity and/or type 2 diabetes mellitus when compared to normal-weight and normo-glycemic individuals [13,14,15]. Despite these consistent findings and despite the high relevance of local and systemic CTRP-3 effects in the regulation of inflammatory and metabolic processes, there is a considerable lack of data in the literature regarding the effects of weight loss on circulating CTRP-3 levels in obesity.

In the present study, we investigated serum CTRP-3 concentrations in two large patient cohorts of severely obese individuals undergoing either bariatric surgery or a low-calorie formula diet. For baseline CTRP-3 concentrations, gender- and T2D-related differences were observed, as described earlier in the literature [13,17,26]. Taken together, CTRP-3 levels are higher in females and lower in patients suffering from T2D and hypertension. Positive correlations of CTRP-3 levels with adiponectin, leptin, and BMI were observed. Of note, serum CTRP-3 and HDL cholesterol levels were positively correlated in patients under LCD, potentially implying a regulatory link between CTRP-3 and cholesterol metabolism. In vitro, simvastatin as an inhibitor of cholesterol synthesis did not affect CTRP-3 gene expression in adipocytes. However, future and more comprehensive approaches in vitro and in vivo will be necessary to clarify this issue. Importantly, gene expression levels of CTRP-3 in both visceral and subcutaneous adipose tissue from bariatric surgery patients were positively correlated with the respective systemic concentrations. Moreover, CTRP-3 mRNA levels were observed to be considerably higher in subcutaneous than in visceral adipose tissue of obese subjects awaiting bariatric surgery. The physiological relevance of this observation has to be investigated in detail in further studies. However, we are convinced that there is some physiological relevance because these expression data fit very well with the observation in our study that systemic CTRP-3 protein concentration is stronger related to subcutaneous than to visceral adipose tissue gene expression. In wildtype mice (C57BL/6), a contrary situation was observed with lower CTRP-3 expression in subcutaneous than in intra-abdominal adipose tissue. Apart from potential species-effects, this discrepancy might be caused by obesity-associated effects which were absent in the lean and metabolically healthy mice.

Analysis of CTRP-3 levels at defined study time-points following bariatric surgery and during the dietary program, respectively, revealed a strong, rapid, and sustained decrease of CTRP-3 serum levels in both cohorts. CTRP-3 concentrations were reduced to about 60% of baseline levels at study point V3 (three months after surgery or start of dietary intervention, respectively). Thus, systemic CTRP-3 appears to be down-regulated within the context of weight loss, a finding that was somewhat surprising due to the inverse relation of CTRP-3 levels and BMI known from the literature [13,14,15]. Reduction of body weight and BMI might have been expected to be accomplished by an increase of CTRP-3. The observed decline is also contrary to the upregulation of circulating adiponectin upon bariatric surgery [27,28], which is closely related to CTRP-3 regarding immuno-metabolic functions and molecular structure. A possible explanation might be given by the rapid and extensive loss of fat mass achieved by both intervention strategies. Since a high proportion of circulating CTRP-3 is secreted by adipose tissue, it is reasonable to assume that an intense reduction of fat mass might result in decreased serum protein levels. Regarding the inverse regulation of adiponectin levels, one might speculate about potential compensatory mechanisms within the remaining adipose tissue, such as increased transcriptional activity of ADIPOQ promoter.

Most interestingly, the rapid decline of CTRP-3 serum levels had immediately started only three days after bariatric surgery (at visit V1), when weight loss had not yet occurred. This finding strongly argues for additional and potent mechanisms acting independent of weight loss. With regard to the immediate and radical anatomical and physiological changes caused by bariatric surgery, it seems reasonable to assume that hormonal (incretins) and metabolic factors (bile acids) downregulate CTRP-3. Since incretin hormones and total bile acids are upregulated rapidly following gastric bypass surgery [20,21,29,30], we set out to investigate bile acid sub-species and incretins in vitro regarding CTRP-3 gene expression. Of note, the primary bile acids cholic acid and chenodeoxycholic acid effectively downregulated CTRP-3 mRNA levels in 3T3-L1 adipocytes. While GLCA inhibited CTRP-3 gene expression in adipocytes in vitro, TDCA and TLCA, as well as the unconjugated secondary bile acid deoxycholic acid, had no significant effects on CTRP-3 mRNA levels. Interestingly, taurohyocholic acid (known to be increased in cholestasis and upon sleeve gastrectomy for obesity) [31] strongly suppressed CTRP-3 expression. Unlike cholic acid or chenodeoxycholic acid, taurohyocholic acid has a third hydroxyl group in the α-conformation at the 6-position. These data clearly argue for differential and very specific effects of bile acids dependent on the molecular structure. Among the incretins, GLP-1 significantly downregulated CTRP-3 expression in adipocytes, whereas GIP caused a non-significant trend in lowering CTRP-3 expression.

Taken together, these findings suggest for the first time that the rapid (thus weight loss-independent) decline of circulating CTRP-3 levels after bariatric surgery might be caused by specific regulatory effects of certain incretins and bile acid sub-species in adipocytes and adipose tissue.

The bile acid receptors FXR and TGR5 are expressed in adipocytes and the evidence of functional bile acid signaling pathways in adipocytes with immuno-modulatory effects has been reported recently by our group [25]. In order to verify the intriguing hypothesis of a regulatory interrelation of CTRP-3 with bile acids in adipocytes and adipose tissue, its impact on gene expression of FXR and TGR5 in adipose tissue was investigated in vivo. In a murine model of adipocyte-specific CTRP-3 knockout, mRNA levels of both receptors were considerably upregulated in intra-abdominal adipose tissue when compared to littermate controls. Contrary to these effects observed in intra-abdominal adipose tissue, bile acid receptor mRNA levels were unaffected by CTRP-3 deficiency in primary adipocytes derived from this tissue and were even decreased in CTRP-3 deficient subcutaneous adipocytes. These findings indicate that deficiency of adipocyte-derived CTRP-3 might rather affect FXR and TGR5 gene expression in non-adipocytic cell-types within adipose tissue, such as cells within the stroma-vascular fraction. 

In a separate experimental setting, intraperitoneal injection of exogenous, recombinant CTRP-3 (from eukaryotic expression system) in C57BL/6 wildtype mice exhibited differential and short-time effects on bile acid receptor expression in intra-abdominal adipose tissue. FXR mRNA levels were significantly downregulated by CTRP-3 application. On the other hand, TGR5 gene expression was observed to be even induced by a high dose of CTRP-3. Apparently somewhat inconsistent at a first glance, this finding might indicate differential mechanisms of regulatory interrelation between CTRP-3 and these two distinct bile acid receptors. Future approaches are necessary with experimental settings including FXR- and TGR5-specific agonists and antagonists as well as comprehensive signal transduction analysis both in vitro and in vivo. Treatment of 3T3-L1 adipocytes with CTRP-3 even with a high dose of 10 µg/mL affected neither FXR nor TGR5 mRNA levels. Since CTRP-3 stimulation of 3T3-L1 adipocytes in vitro does not affect TGR5 or FXR expression, the context of other cell types (fibroblasts, mesenchymal stem cells, smooth muscle cells, endothelial cells, stroma-vascular cells) residing in total adipose tissue might be responsible for co-modulating the effects of CTRP-3 on bile acid receptors in adipose tissue.

## 4. Materials and Methods

### 4.1. The ROBS Study Cohort (Research in Obesity and Bariatric Surgery)

The ROBS study cohort has been introduced recently, more detailed information concerning outline and study characteristics can be retrieved from the literature [23]. Briefly, serum samples and specimens from subcutaneous (abdominal) and visceral (intra-abdominal) adipose tissue were collected from the ROBS study cohort which represents an open-label, non-randomized, prospective, and observational (explorative and confirmatory) study of patients routinely undergoing either bariatric surgery (gastric sleeve or Roux-en-Y gastric bypass; *n* = 179) or a low-calorie formula diet (*n* = 131) in a single tertiary care center at the University of Giessen, Germany. Only compliant patients without any evidence of depression or mental illness who had successfully taken part in a professional conservative weight reduction program in the past were admitted for surgery. Patients were treated by a multidisciplinary team of physicians and professionals from Internal Medicine, Endocrinology/Diabetology, Metabolic/Visceral Surgery, Psychosomatic Medicine/Psychotherapy, Nutritional Science/Dietetics, and Sports Medicine at the Obesity Center at the University of Giessen, Germany. The study was approved by the local ethical committee at the University of Giessen, Germany (Ethik-Kommission am Fachbereich Medizin, 12.06.2014, identification code: 101/14). All patients gave informed consent and were informed about the aim of the study. Data anonymization and privacy policy were accurately applied.

Obese patients with a body mass index (BMI) ≥ 40 kg/m^2^ or with a BMI ≥ 35 kg/m^2^ and coexisting type 2 diabetes were consecutively admitted for bariatric surgery from January 2015 up to now. Exclusion criteria were: pregnancy, evidence of or suspicion on underlying endocrine diseases, untreated bulimia nervosa and binge eating behavior, use of illicit drugs, neoplasm, severe psychiatric disorders, psychosis, and psychopathologic instability.

### 4.2. Serum Measurement of CTRP-3 Concentrations

The concentrations (ng/mL) of serum CTRP-3 in each sample (visits: V0, V1, V3, V6, V12, V18, and V24) were measured in duplicates by ELISA (DuoSet ELISA development systems, R&D Systems, Wiesbaden, Germany) and are expressed as mean ± standard deviation. Concerning the ELISA measurements, there was an interval of intra-assay variation of 1.5 to 10%. Measurement was generally repeated for samples exceeding a duplicate variation of 20%. The lower detection limit was 78.1 pg/mL.

### 4.3. Cell Culture Experiments

3T3-L1 fibroblasts (pre-adipocytes) [32] were cultured at 37 °C and 5% CO2 in DMEM (Dulbecco’s Modified Eagle Medium, Biochrom AG, Berlin, Germany) supplemented with 10% newborn calf serum (NCS, Sigma-Aldrich, Deisenhofen, Germany) and 1% penicillin/streptomycin (PAN, Aidenbach, Germany). Cells were differentiated into adipocytes at confluence by DMEM/F12/glutamate medium (Lonza, Basel, Switzerland), supplemented with 20 µM 3-isobutyl-methyl-xanthine (Serva, Heidelberg, Germany), 1 µM corticosterone, 100 nM insulin, 200 µM ascorbate, 2 µg/mL transferrin, 5% fetal calf serum (FCS, Sigma-Aldrich, Deisenhofen, Germany), 1 µM biotin, 17 µM pantothenate, 1% penicillin/streptomycin (all from Sigma Aldrich, Deisenhofen, Germany), and 300 µg/mL Pedersen-fetuin (MP Biomedicals, Illkirch, France) [33,34] for 9 days using a slightly modified protocol as reported in the literature [32,35,36,37,38]. Phenotype was controlled by light-microscopy (appearance of extensive accumulation of lipid droplets). Mature adipocytes at day 9 of differentiation were used for stimulation experiments under serum-free culture conditions. The primary bile acids cholic acid (CA; 100 µM) and chenodeoxycholic acid (CDCA; 10 µM) and the secondary bile acids deoxycholic acid (DCA; 10 µM), ursodeoxycholic acid (UDCA; 50 µM) taurodeoxycholic acid (TDCA; 100 µM), glycolithocholic acid (GLCA; 10 µM), taurolithocholic acid (TLCA; 10 µM) (all from Sigma Aldrich, Deisenhofen, Germany), and taurohyocholic acid (THCA; 10 µM) (from Cayman Chemical, Ann Arbor, MI, USA) were used for stimulation experiments (incubation time 18 h). Applied bile acid concentrations had been tested for cytotoxicity in 3T3-L1 cells before [25]. 3T3-L1 adipocytes were treated with the incretins GLP-1 (100 nM), GIP (100 nM), and the cholesterol synthesis inhibitor simvastatin (0.1, 1, and 10 µM) (all from Sigma Aldrich, Deisenhofen, Germany) for 18 h. In order to exclude any unexpected effects on cell viability, LDH (lactate dehydrogenase) activity was generally measured in the supernatants of stimulated cells (Cytotoxicity Detection Kit, Roche, Mannheim, Germany).

### 4.4. Adipose Tissue and Adipocyte mRNA Extraction

Total RNA was isolated from human visceral and subcutaneous adipose tissue using TRIzol^®^-Reagent (Life Technologies GmbH, Darmstadt, Germany) in combination with gentleMACS dissociator and M-tubes (Miltenyi Biotec GmbH, Bergisch Gladbach, Germany) for dissociation. RNA was isolated from prepared tissue using RNeasy^®^ Mini Kit (Qiagen, Hilden, Germany) including DNase digestion (RNase-Free DNase Set, Qiagen, Hilden, Germany) and gene expression was analyzed by reverse transcription of 300 ng RNA (QuantiTect Reverse Transcription Kit from Qiagen, Hilden, Germany) and subsequent real-time PCR (RT-PCR) (iTaq Universal SYBR Green Supermix, CFX Connect RT-PCR system; Bio-Rad, Munich, Germany) of the corresponding cDNA as mentioned below in detail.

### 4.5. Real-Time PCR Analysis of CTRP-3 and Bile Acid Receptor mRNA Expression

Gene expression of CTRP-3 and bile acid receptors in human subcutaneous and visceral adipose tissue depots was analyzed by reverse transcription of isolated RNA and subsequent RT-PCR. The following primer sequences were used:

Human CTRP-3: 5′-GCCCCAGTATCAGGTGTGTA-3′/5′-GCAAAGGTGGAGAAGCGTTG-3′;
Murine CTRP-3: 5′-GGGGTTCTTTATGGAGCATT-3′/5′-AAATGCATCCTTTGAGGTGA-3′;
Human TGR5: 5′-CACTGTTGTCCCTCCTCTCC-3′/5′-ACACTGCTTTGGCTGCTTG-3′;
Murine TGR5: 5′-GAGCGTCGCCCACCACTAGG-3′/5′-CGCTGATCACCCAGCCCCATG-3′;
Murine FXR I (for adipocyte mRNA): 5′-TGGGCTCCGAATCCTCTTAGA-3′/5′-TGGTCCTCAAATAAGATCCTTGG-3′;
Murine FXR II (for adipose tissue mRNA): 5′-GTACAAGTGTAAGAACGGGG-3′/5′-CTTGGTTGTGGAGGTCACTT-3′.

Expression levels of target genes were normalized to gene expression of GAPDH using the primer-pairs 5′-GAGTCCACTGGCGTCTTCAC-3′/5′-CCAGGGGTGCTAAGCAGTT-3′ (human) and 5′-TGTCCGTCGTGGATCTGAC-3′/5′-AGGGAGATGCTCAGTGTTGG-3′ (murine). 2^−ΔΔCT^ method was applied in order to determine relative expression levels of target genes normalized to GAPDH expression. All oligonucleotides used were purchased from Metabion, Martinsried, Germany.

### 4.6. Animals

#### 4.6.1. Tissues from Wildtype and CTRP-3 Knockout Mice

Wildtype (C57BL/6) and transgenic mice with an adipocyte-specific CTRP-3 knockout (full nomenclature: B6NTac.Cg-C1qtnf^3tm3113Arte^Tg(Fabp4-cre)1Rev; abbreviation: CTRP-3 KO) together with litter-mate control mice (B6NTac.Cg-C1qtnf^3tm3113Arte^) were bred under standard conditions and were euthanized for organ and tissue resection. Intra-abdominal and subcutaneous (pooled from whole dorsal and lateral body region’s fat depots) adipose tissue specimens were resected. Specimens were either used for primary cell isolation or were immediately shock-frosted in liquid nitrogen. Adipocyte-specific deletion of exon 4 and subsequent frame-shift mutations within the C1qtnf3 gene were introduced applying the Cre/loxP system, resulting in a dysfunctional gene product. Cell-type specificity of the knockout was achieved by transcriptional control of the Cre recombinase encoding transgene by the adipocyte-specific aP2-promotor (from Fabp4 gene). B6NTac.Cg-C1qtnf^3tm3113Arte^ mice carrying C1qtnf3 gene alleles with loxP-flanked exon 4 were created in collaboration with Taconic Artemis (Cologne, Germany). B6.Cg-Tg(Fabp4-cre)1Rev/J mice (purchased from Jackson Laboratories) were back-crossed to C57BL/6NTac genetic background applying the Speed Congenics method for at least 6 generations, resulting in the strain B6.Cg-Tg(Fabp4-cre)1Rev/N. This offspring was crossed with C57BL/6NTac-C1qtnf^3tm3113Arte^ mice to generate B6NTac.Cg-C1qtnf^3tm3113Arte^Tg(Fabp4-cre)1Rev mice with adipocyte-specific knockout of CTRP-3 (CTRP-3 KO).

#### 4.6.2. Isolation and Cell Culture of Primary Murine Adipocytes

For primary cell culture, fresh intra-abdominal and subcutaneous adipose tissue obtained from CTRP-3 KO mice was cut into small pieces and treated with 0.225 U/mL collagenase NB6 (from Serva, Heidelberg, Germany) at 37 °C for a maximum of 60 min. Digestion process was stopped by adding twice the amount of buffer (PBS containing 0.5% BSA and 2 mM EDTA; from Sigma Aldrich, Deisenhofen, Germany). Cell suspension was filtered by 120 µm nylon mesh to eliminate undissolved tissue. Pre-adipocytes were separated from adipocytes by 10 min centrifugation at 300 g and 4 °C. Magnetic labeling plus depletion of non-adipocyte progenitor cells was done according to the manufacturer’s instructions (Adipose Tissue Progenitor Isolation Kit mouse, MACS Miltenyi Biotec, Bergisch Gladbach, Germany), as well as magnetic labeling and positive selection of adipocyte progenitor cells. Isolated pre-adipocytes were seeded at a density of 2.03 × 104 cells/cm² in DMEM (Dulbecco´s Modified Eagle Medium, Biochrom AG, Berlin, Germany) which was supplemented with 10% newborn calf serum (NCS; from Sigma-Aldrich, Deisenhofen, Germany) and cultured at 37 °C and 5% CO2. Adipocyte differentiation was initiated after cells reached 85% confluency. Media for hormonal differentiation were supplemented as described above for 3T3-L1 cell-line. Cell phenotype during adipocyte differentiation was monitored by light microscopy.

#### 4.6.3. Intraperitoneal Application of Recombinant CTRP-3 in Mice

After an overnight fast, C57BL/6 mice (male, 10 weeks old, weight 24–28 g, Charles River, Sulzfeld, Germany) were treated with an intraperitoneal (i.p.) injection of sterile PBS as a control or with recombinant CTRP-3 (10 µg/animal; expressed in H5 cells (Invitrogen, Karlsruhe, Germany) applying BacPak Baculovirus Expression System (BD Biosciences, Palo Alto, CA, USA)). Intra-abdominal adipose tissue specimens were resected post mortem and were shock-frosted in fluid nitrogen for further analysis of bile acid receptor mRNA expression. Animal experiments were performed at the University of Regensburg, Germany, and this animal study was approved by the local government agency (Regierungspraesidium Oberpfalz, No. 54-2532.1-14/10).

#### 4.6.4. Data Base and Statistical Analysis

The ROBS database represents a relational database management program. For statistical analysis, data were exported into the statistical software program *SPSS 26.0* (IBM, Armonk, NY, USA). CTRP-3 serum concentrations and gene expression levels did not follow a Gaussian distribution. Non-parametric numerical parameters were analyzed by the Mann–Whitney U-test (for 2 unrelated samples), the Kruskal–Wallis test (>2 unrelated samples), the Wilcoxon test (for 2 related samples) or the Friedman test (>2 related samples). Correlation analysis was performed by using the Pearson test (parametric parameters) or the Spearman test (non-parametric parameters). Partial correlation analysis was applied to control for possible covariates. A p-value below 0.05 (two tailed) was considered as statistically significant. Bar diagrams indicate means ± SEM (standard error of the mean). Box plots are shown indicating median, upper/lower quartiles, interquartile range, minimum/maximum values, and outliers.

## 5. Conclusions

The present study reports a highly significant decrease of circulating CTRP-3 levels by either surgical or dietary intervention, indicating a general, negative regulation of systemic CTRP-3 during weight loss. Among patients undergoing bariatric surgery, this decline occurred very rapidly and sustainable upon the intervention. This rapid decline suggests weight loss-independent and alternative mechanisms that down-regulate CTRP-3. Considering the changes of gastro-intestinal anatomy introduced by bariatric surgery and the well-known increase of circulating incretins and bile acids, potential regulatory interactions of CTRP-3 with these metabolic factors were investigated and proven to play a role in vitro. GLP-1 as well as primary bile acids CA and CDCA down-regulated CTRP-3 mRNA levels in adipocytes, whereas other bile acids did not exhibit significant effects. Most interestingly and in accordance with these findings, gene expression of bile acid receptors FXR and TGR5 was observed to be elevated in murine adipose tissue derived from animals with adipocyte-specific CTRP-3 knockout. Taken together, the present data strongly argue for weight loss-dependent and weight loss-independent (GLP-1, primary bile acids) mechanisms regulating CTRP-3.

## Figures and Tables

**Table 1:** Correlation analysis (Spearman rho test) of serum C1q/TNF-related protein-3 (CTRP-3) concentrations. (**A**) Significant correlations of basal serum CTRP-3 levels with gene expression and adipokines in severely obese patients at the time point of bariatric surgery (BS) before weight loss (asservation of subcutaneous (sc) and visceral (vis) adipose tissue) (*n* = 179). (**B**) Significant correlations of basal serum CTRP-3 levels with adipokines in patients starting low calorie diet (LCD; no asservation of adipose tissue) (*n* = 131).

**A—Correlation Analysis of**	**rho**	***p***
Serum CTRP-3 concentrations in BS patients with		
CTRP-3 gene expression in sc AT	+0.23	0.003
CTRP-3 gene expression in vis AT	+0.16	0.033
Adiponectin (µg/ml)	+0.38	<0.001
Leptin (ng/ml)	+0.16	0.033
**B—Correlation Analysis of**	**rho**	***p***
Serum CTRP-3 concentrations in LCD patients with		
Adiponectin (µg/mL)	+0.32	<0.001
Leptin (ng/mL)	+0.23	0.007

## Appendix A

**Table A1:** Human CTRP-3 serum levels in obesity as measured by different ELISA kit suppliers. Serum data from the present study (human CTRP-3 ELISA Kit from R&D Systems, Wiesbaden, Germany) are depicted together with data published by Deng et al. (*Diabetol Metab. Syndr.*, **2015** [14]; ELISA Kit from Aviscera Bioscience, Santa Clara, CA, USA), Wolf et al. (*PLoS ONE*, **2015** [13]; ELISA Kit from AdipoGen, Incheon, Korea), and Jain et al. (*Int. J. Endocrinol. Metab.*, **2019** [24]; ELISA Kit from Wuhan EIAab Science Co.). BS, bariatric surgery (present study); LCD, low calorie diet (present study).

Cohort	Mean BMI (kg/m^2^)	Mean CTRP-3 Serum Concentrations (ng/ml)	ELISA Supplier	Study	Reference
Bariatric surgery (*n* = 179)	53.3 ± 6.9	106.5 ± 51.0	CTRP-3 DuoSet ELISA, R&D system, Wiesbaden, Germany	Schmid, A. et al.	present
Low calorie diet (*n* = 131)	43.5 ± 6.7	108.1 ± 40.5	CTRP-3 DuoSet ELISA, R&D system, Wiesbaden, Germany	Schmid, A. et al.	present
Obesity subgroup (*n* = 180)	28.02 ± 1.65	94.53 ± 43.94	CTRP-3 ELISA Aviscera Bioscience, Santa Clara, CA, USA	Deng, W. et al.	[14]
Obesity subgroup (*n* = 44)	45.2 ± 1.1	405 ± 8.3	CTRP-3 ELISA AdipoGen, Incheon, Korea	Wolf, R.M. et al.	[13]
Obesity subgroup (*n* = 30)	33.7 ± 4.4	258.22 ± 147.62	CTRP-3 ELISA Wuhan El Aab Science Co.	Jain, N.B. et al.	[24]
